# Comparison of ROX and HACOR scales to predict high-flow nasal cannula failure in patients with SARS-CoV-2 pneumonia

**DOI:** 10.1038/s41598-021-02078-5

**Published:** 2021-11-19

**Authors:** Carlos Fernando Valencia, Oscar David Lucero, Onofre Casas Castro, Andrey Alexandrovich Sanko, Peter Alfonso Olejua

**Affiliations:** 1 Emergency Unit, Hospital Universitario San Ignacio, Pontificia Universidad Javeriana, Carrera 7 No. 40-62, Bogotá, Colombia; 2 Internal Medicine Resident, Hospital Universitario San Ignacio, Pontificia Universidad Javeriana, Carrera 7 No. 40-62, Bogotá, Colombia; 3 Emergency Medicine Resident, Hospital Universitario San Ignacio, Pontificia Universidad Javeriana, Carrera 7 No. 40-62, Bogotá, Colombia; 4 General Medicine, Pontificia Universidad Javeriana, Carrera 7 No. 40-62, Bogotá, Colombia; 5 Epidemiology Department, Pontificia Universidad Javeriana, Carrera 7 No. 40-62, Bogotá, Colombia

**Keywords:** Viral infection, Epidemiology

## Abstract

The pandemic of SARSCov2 infection has created a challenge in health services worldwide. Some scales have been applied to evaluate the risk of intubation, such as the ROX and HACOR. The objective of this study is to compare the predictive capacity of the HACOR scale and the ROX index and define the optimal cut-off points. Study of diagnostic tests based on a retrospective cohort. Composite outcome was the proportion of patients that needed endotracheal intubation (ETI) or died of COVID19 pneumonia. Discrimination capacity was compared by the area under the curve of each of the two scales and the optimal cut-off point was determined using the Liu method. 245 patients were included, of which 140 (57%) required ETI and 152 (62%) had the composite end result of high-flow nasal cannula (HFNC) failure. The discrimination capacity was similar for the two scales with an area under receiver operating characteristic curve of 0.71 and 0.72 for the HACOR scale for the ROX index, respectively. The optimal cut-off point for the ROX index was 5.6 (sensitivity 62% specificity 65%), while the optimal cut-off point for the HACOR scale was 5.5 (sensitivity 66% specificity 65%). The HACOR scale and the ROX index have a moderate predictive capacity to predict failures to the HFNC strategy. They can be used in conjunction with other clinical variables to define which patients may require invasive mechanical ventilation.

## Introduction

Perhaps the most feared consequence of COVID-19 infection is the development of severe disease which may lead to intensive care unit (ICU) admission. As shown in Hu et al. systematic review and meta analysis, incidence of Acute Respiratory Distress Syndrome (ARDS) ranges from 5.6 to 13.2%, Acute Cardiac Injury reaches 5.8%, shock incidence reaches 4.7% and Acute Kidney Injury (AKI) 2.1%, all of which are important determinants and contributors to an overall ICU admission close to 20% of severe COVID-19 cases^1^. Another study reported 16% of patients with severe COVID-19 pneumonia required ICU admission^2^.

Among patients admitted to the ICU, the rate of invasive mechanical ventilation (IMV) ranges between 71 and 90%^3^, with mortality close to 50%^4,5^. There are different ways to deliver oxygen in patients with severe pneumonia such as high-flow nasal cannula (HFNC), standard oxygen therapy delivered through a face mask, or non-invasive mechanical ventilation (NIMV). Although there was no significant difference between the requirement for endotracheal intubation (ETI) in the different interventions, the number of ventilator-free days at day 28 was significantly higher in the high-flow-oxygen group^6^. Therefore, the use of HFNC associated with the prone position^7,8^ is a widely used strategy for severe COVID-19 pneumonia as first-line treatment, since some patients can improve oxygenation and fatigue and avoid invasive mechanical ventilation with these strategies^9^.

However, it is also known that the delay initiation of IMV in patients with severe pneumonia leads to an increase in mortality^10^, being the reason why it is so important to identify in a timely manner patients in whom this therapy may fail. To do this, scales have been created to predict failure to NIMV strategies, including the ROX index, which consists of a relationship between arterial oxygen saturation/fraction of inspired oxygen ratio (SpO2/FIO2) and respiratory frequency. It was validated in 2016^11^, as an index for predicting HFNC therapy failure in patients with severe pneumonia. Roca et al.^6^ found that a score of 4.9 after 2 h of HFNC therapy predicts failure to HFNC (AUROC of 0.74). In addition, studies have been done that validate its usefulness in patients with COVID19 pneumonia^12–14^. However, the main problem with the ROX scale lies in the delay in the initiation of IMV in people who require it.

In 2017, Duan et al.^10^ created a tool called HACOR, an acronym for heart rate, acidosis, state of consciousness, oxygenation, and respiratory rate (RR), to predict NIMV failure. In 2020, Carrilo et al.^12^ validated this tool with 2711 patients, showing that the HACOR scale accurately predicted NIMV failure in the first hour. A cut-off value of 8 points at 1 h of HFNC beginning had an AUROC greater than 0.9 for predicting NIMV failure in patients with pneumonia and adult respiratory distress syndrome (ARDS). To our knowledge, the HACOR score has not been validated in patients with COVID19.

Although these scales have been validated in patients with severe pneumonia, we do not know if the HACOR scale is useful in patients with COVID19 and if its discrimination capacity is better than the ROX scale. The objective of this study is to compare the discrimination capacity between the HACOR and ROX scales to predict the risk of IMV in patients with severe SARSCOV2 pneumonia who are with HFNC strategy in the resuscitation unit of a hospital in Colombia.

## Methods

### Trial oversight

Study of diagnostic tests based on a retrospective cohort. We conducted the study in the emergency unit of a high complexity University Hospital in Bogota, Colombia. The study protocol was approved by the “Research and Institutional Ethics Committee of the faculty of medicine of the Pontificia Universidad Javeriana, and the Hospital Universitario San Ignacio”. Since the study was based on data collection, patient consent was not required. This waiver is given in agreement with the Helsinki declaration and resolution number 008430 of 1993 issued by the Ministry of Health from the Republic of Colombia.

The first investigator was responsible for searching the hospital’s database, while the second investigator ensured adherence to the protocol. Tests were conducted in accordance with principles of the Helsinki declaration. Authors assume responsibility for the integrity of the data and its analysis.

### Patients

Total adult population identified with viral pneumonia by SARS CoV2 that required HFNC in the emergency department of the San Ignacio Hospital, between august and December of 2020 were eligible for enrollment. Data were taken from patients older than 18 years of age who had the diagnosis of SARS CoV2, confirmed by polymerase chain reaction (PCR). Patients with SARS CoV2 pneumonia who had required immediate ventilatory support with invasive mechanical ventilation and patients with severe or refractory agitation to sedation, massive bronchial aspiration, inability to manage bronchial secretions were excluded. Patients eligible for HFNC but with a signed will not to proceed to HFNC were also excluded.

### Trial procedures

The decision to initiate a HFNC was made by the emergency physician or internist attending the patient, based on the following criteria: clinical presentation of respiratory failure (such as the use of accessory muscles, breathing rate greater than 20 respirations per minute, paradoxical abdominal movement, desaturation despite nasal cannula or venturi therapy), partial pressure of arterial oxygen (PaO2) less than < 60 mmHg or the PaO2/inspired fraction oxygen (FiO2) ratio < 300 with supplemental oxygen. In the emergency department of the San Ignacio Hospital, FiO2 ratio is currently calculated using the 3% formula [21% + (oxygen flow rate in L/min × 3)], which has been shown to have the highest accurate estimation of FiO2 in the literature^15^.

HFNC was managed by respiratory therapist professionals and patient’s emergency physicians and internists, depending on the case. Masks, heated humidifiers, air–oxygen blenders, and cannulas (i.e., consumable materials) were owned by the Hospital. The device used as high-flow therapy was the Optiflow™ and the parameters at the beginning of the therapy were established according to the patient’s last partial PaO2/FiO2 to achieve a SaO2 greater than 90%. Once respiratory failure improved, progressive decrease in the parameters of the HFNC were initiated. This was done when the patient had a breathing rate of fewer than 25 breaths per minute, without signs of muscle fatigue, had a SaO2 greater than 90%, and PaO2/FiO2 in ascent concerning that taken before the beginning of the HFNC. The inspired fraction of oxygen parameter was lowered initially and, according to the patient’s tolerance, the flow parameter was subsequently lowered. All patients underwent gasometric monitoring before the onset of HFNC.

Every patient selected for our study was given HFNC therapy trial when considered necessary by the physician, there was no use of CPAP or other non invasive high flow oxygen therapy. The need to proceed to IMV was determined by the physician after a minimum of 2 h period and after a complete assessment of vital signs, arterial blood gas work and calculation of HACOR and ROX scales were performed. These procedures were done in a consistent basis independent of the specialist (emergency or internal medicine physician). If no improvement was noticed in each of the clinical (signs of muscle fatigue, SaO2 greater than 90%) and lab variables (PaO2/FiO2 ascent), ETI was defined.

The definition of coinfection was that of the Centers for Disease Control and Prevention (US)^16^, which defines coinfection as one occurring concurrently with the initial infection. The diagnosis of coinfection was made in people with clinical and radiological criteria that were not compatible with COVID19 infection (for example, chest computed tomography that did not present a classic pattern of COVID19 infection, together with the microbiological finding by culture tests and polymerase chain reaction (PCR) taken from sputum or endotracheal aspirate within 48 h of admission.

### Outcomes

Primary outcome was HFNC therapy failure, including need for mechanical ventilation onset and death associated with COVID-19 pneumonia.

### Statistical analysis

Demographic and epidemiological characteristics of patients diagnosed with COVID-19 pneumonia who required HFNC therapy were described.

According to the recommendations of the Transparent reporting of a multivariable prediction model for Individual prognosis or diagnosis statement (TRIPOD)^17^, the minimum sample size was 100 events and 100 not events, so a minimum of 100 failures to the HFNC was sought. The baseline characteristics were compared between the treatment groups with the use of chi-square and Wilcoxon tests.

The discrimination capacity of the HACOR and ROX scales was evaluated using the area under the receiver operating characteristic curve (AUROC). The comparison of both scales was carried out by comparing the areas under the curve of the two scales. A secondary analysis of the ROX and HACOR scales was finally performed to predict failure of HFNC.

Finally, the Liu method was performed to identify the cut-off point with the best performance of the HACOR and ROX scales^18^.

## Results

### Patient characteristics

A total of 245 patients were included in the study. Most of them were men (65%). Mean (± SD) age of the patients was 62 (± 13) years. A total of 93 patients (38%) were 18–60 years of age and 152 (62%) were 60 years of age or older. 152 (62%) patients had the final composite outcome of HFNC failure (requirement of ETI or death by COVID19). Death associated with SARSCov2 infection occurred in 72 patients (29%).

Table 1 shows key characteristics of the patients, stratified into two groups according to whether the patient had the final composite outcome. Interestingly, there were clinically significant imbalances in baseline characteristics with respect to prior history of hypertension, atrial fibrillation, and chronic kidney disease (CKD) between both groups.

**Table 1:** Characteristics of patients according to HFNC failure (final composite outcome including requirement of endotracheal intubation or death by COVID19).

Characteristic	Failure to HFNC		
	Yes n = 152	No n = 93	*p* value
Age, years Mean (SD)	64.16 (13.0)	59.88 (14.8)	0.040
Age group, n (%)			0.13
< 40	6 (4)	11 (12)	
40–59	47 (31)	29 (31)	
60–79	83 (55)	46 (50)	
≥ 80	16 (11)	7 (8)	
Sex male, n (%)	106 (70)	54 (58)	0.073
BMI^†^ mean (SD)	28.1 (5.3)	27.2 (5.0)	0.080
Diabetes mellitus, n (%)	52 (34)	26 (28)	0.33
Atrial fibrillation, n (%)	17 (11)	2 (2)	0.012
CKD, n (%)	21 (14)	4 (4)	0.017
Heart failure, n (%)	21 (14)	5 (5)	0.053
ACS antecedent, n (%)	11 (7)	4 (4)	0.42
Hypertension, n (%)	90 (59)	38 (41)	0.006
Isolation (bacterial or viral different from SARSCoV2), n (%)	51 (34)	5 (6)	< 0.0001
COPD, n (%)	21 (14)	17 (18)	0.37
CVA/TIA, n (%)	2 (1)	3 (3)	0.37
Dementia, n (%)	2 (1)	1 (1)	0.9999
Cirrhosis, n (%)	1 (1)	1 (1)	0.9999
Autoimmune disease, n (%)	4 (3)	4 (4)	0.48
Solid tumors, n (%)	12 (8)	4 (4)	0.42
Hematologic malignancy, n (%)	2 (1)	1 (1)	0.9999
HIV, n (%)	0 (0)	1 (1)	0.38
Heart rate, Mean (SD)^a^	85 (16)	80 (15)	0.014
Glasgow coma score < 15, n (%)^a^	55 (36)	8 (9)	< 0.0001
Respiratory rate, Mean (SD)^b^	26 (5.7)	22 (3.5)	< 0.0001
HACOR, Mean (SD)^b^	7.14 (3.6)	4.45 (2.2)	< 0.0001
ROX Index, Mean (SD)^b^	5.61 (4.1)	6.63 (1.9)	< 0.0001
Death by SARS-CoV 2, n (%)	72 (47)	0 (0)	< 0.0001
Required OTI, n (%)	140 (92)	0 (0)	< 0.0001

*SD* standard deviation, *BMI* body mass index, *DM* diabetes mellitus, *CKD* chronic kidney disease, *HFNC* high-flow nasal cannula, *ACS* acute coronary syndrome, *COPD* chronic obstructive pulmonary disease, *CVA* cerebrovascular accident, *TIA* transient ischemic attack, *HIV* human immunodeficiency virus, *ETI* endotracheal intubation.

There were no significant differences (*p* < 0.05) between the study groups.

^a^Before Introduction of HFNC.

^b^2 h post HFNC.

^†^The body-mass index is the weight in kilograms divided by the square of the height in meters.

In the HFNC failure group, the median age was 64 years (*p* = 0.04). Also, In the group with HFNC failure, there was a significantly higher number of people with atrial fibrillation, chronic kidney disease and hypertension. Of the clinical and paraclinical variables taken before the introduction of HFNC, the blood pressure of Co2 (PaCo2) (29.8 vs. 31.4, *p* = 0.014) and the Glasgow coma scale < 15 (*p* < 0.0001) were statistically significant between the groups that did not fail and those that did fail the HFNC (Table 1). There were statistically significant differences at 2 h after starting the HFNC strategy in the variables RR (25 vs. 22 breaths per minute *p* < 0.0001) (Table 1), pH (7.43 vs. 7.46, *p* = 0.004), SaO2 (92% vs. 95%, *p* < 0.0001), PaO2 (78.1 vs. 89.0, *p* < 0.0001), PaO2/Fio2 (103.6 vs. 133.2, *p* < 0.0001) (Table 2). The 2-h ROX index was 6.6 and 5.6 for patients who did not fail and failed to HFNC, respectively (*p* < 0.0001). The HACOR score was 4.45 and 7.14 for patients who did not fail and failed to HFNC at 2 h, respectively (*p* < 0.0001). The mean time to failure of the HFNC was 1.4 days (± 3.8).

**Table 2:** Basal and two hours arterial gases after initiation of the HFNC, distributed according to HFNC failure (final composite outcome including requirement of endotracheal intubation or death by COVID19).

Characteristic	Failure to HFNC		
	Yes	No	*p* value
pH^a^, Mean (SD)	7.45 (± 0.05)	7.45 (± 0.05)	0.20
PaO2^a^, Mean (SD)	72.5 (25.0)	73.1 (20.8)	0.22
PaCO2^a^, Mean (SD)	29.8 (6.4)	31.4 (7.2)	0.014
PaO2/Fio2^a^, Mean (SD)	111.6 (46.8)	121.8 (51.1)	0.16
SaO2^b^, Mean (SD)	92 (6.5)	95 (3.0)	< 0.0001
pH^b^, Mean (SD)	7.43 (0.08)	7.46 (0.05)	0.004
PaO2^b^, Mean (SD)	78.1 (23.2)	89.0 (28.3)	< 0.0001
PaCO2^b^, Mean (SD)	34.2 (10.9)	32.7 (7.1)	0.80
PaO2/Fio2^b^, Mean (SD)	103.6 (38.6)	133.2 (50.9)	< 0.0001

*SD* standard deviation, *HFNC* high-flow nasal cannula, *SaO2* blood–oxygen saturation, *PaO2* arterial partial pressure of oxygen, *PaCO2* partial pressure of carbon dioxide, *Fio2* fraction of inspired oxygen.

There were no significant differences (*p* < 0.05) between the study groups.

^a^Before HFNC.

^b^2 h post HFNC.

In univariate analysis (Table 3) the variables that were associated with the worst outcome were HACOR score (OR 1.33, CI 1.10–1.63, *p* = 0.004), ROX index (OR 0.86, CI 0.76–0.98, *p* = 0.025) male sex (OR 2.37, CI 1.10–5.27, *p* = 0.031), chronic kidney disease (OR 4.61, CI 1.25–21.11, *p* = 0.032), RR prior to the onset of HFNC (OR 1.15, CI 1.05–1.27, *p* = 0.002) and the presence of a viral or bacterial infection different from SarsCov (OR 11.20, CI 4.28–29.27, *p* < 0.001). However, in the multivariate reduction model the variables that were associated with the worst outcome were the HACOR score (OR 1.43, CI 1.25–1.67, *p* < 0.001),the history of chronic kidney disease (OR 3.90, CI 1.24–15.19, *p* = 0.029), arterial hypertension (OR 2.46, CI 1.29–4.81, *p* = 0.007), respiratory rate prior to the onset of HFNC (OR 1.14, CI 1.05–1.23, *p* = 0.001), the PaCo2 taken prior to the initiation of HNFC (OR 0.93, CI 0.88–0.98, *p* = 0.005) and the presence of a viral or bacterial infection different from SarsCov (OR 15.23, CI 5.15–45.04, *p* < 0.001).

**Table 3:** Univariate and multivariate analysis of factors associated with the final composite outcome (requirement of endotracheal intubation or death by COVID19).

Predictors	Univariate analysis			Multivariate analysis		
	Odds ratios	CI	*p*	Odds ratios	CI	*p*
ROX Index	0.86	0.76–0.98	0.025	0.99	0.90–1.09	0.947
HACOR	1.33	1.10–1.63	0.004	1.43	1.25–1.67	< 0.001
Sex male	2.37	1.10–5.27	0.031			
Age	1.03	0.99–1.06	0.108			
BMI^**†**^	1.04	0.96–1.12	0.367			
Atrial fibrillation	3.70	0.73–29.34	0.151			
CKD	4.61	1.25–21.11	0.032	3.90	1.24–15.19	0.029
Hypertension	2.01	0.97–4.25	0.063	2.46	1.29–4.81	0.007
COPD	0.37	0.13–0.98	0.047			
pH before HFNC	11.00	0.00–70,685.37	0.589			
PaO2 before HFNC	1.01	1.00–1.03	0.183			
PaCO2 before HFNC	0.95	0.88–1.02	0.173	0.93	0.88–0.98	0.005
PaO2/Fio2 before HFNC	1.00	0.99–1.01	0.750			
Glasgow coma score^a^	0.63	0.30–1.17	0.175			
Heart rate^b^	1.01	0.99–1.04	0.301			
Respiratory rate^b^	1.15	1.05–1.27	0.002	1.14	1.05–1.23	0.001
Observations	230	230				
*R*^2^ Tjur	0.344	0.285				

There were no significant differences (*p* < 0.05) between the study groups.

*BMI* body mass index, *CKD* chronic kidney disease, *COPD* chronic obstructive pulmonary disease, *HFNC* high-flow nasal cannula, *SaO2* blood–oxygen saturation, *PaO2* arterial partial pressure of oxygen, *PaCO2* partial pressure of carbon dioxide, *Fio2* fraction of inspired oxygen.

^a^Before introduction of HFNC.

^b^2 h post HFNC.

^†^The body-mass index is the weight in kilograms divided by the square of the height in meters.

**Figure 1:**
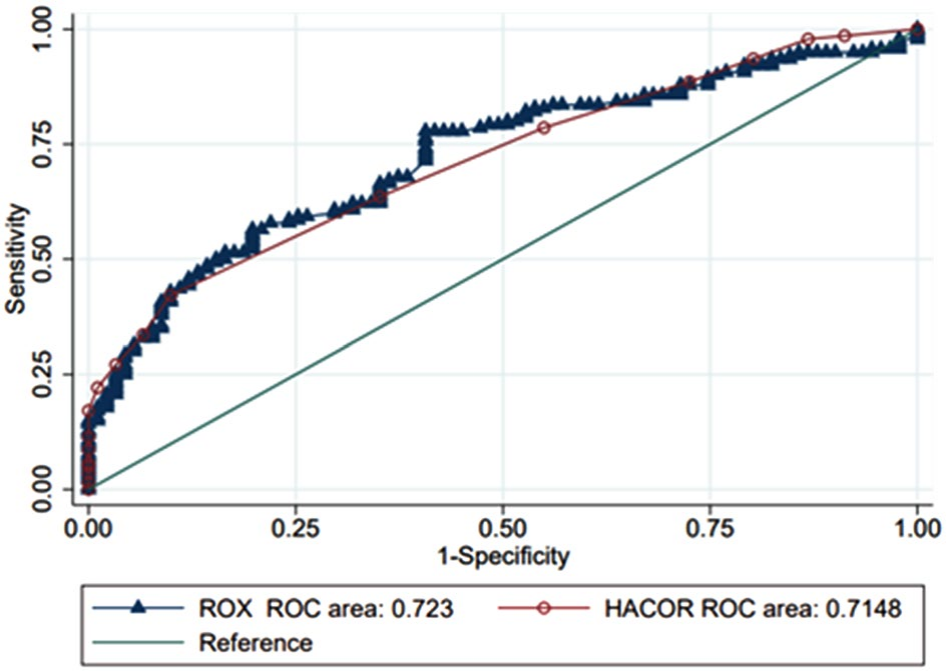
AUROC for HACOR and ROX scores in predicting the final composite outcome (requirement of endotracheal intubation or death by COVID19) after 2 h of High flow nasal cannula. ROX ROC: 0.72 sensitivity 62% specificity 65% CI 0.66–0.79. HACOR ROC: 0.71 sensitivity 66% specificity 65% CI 0.65–0.78.

In the univariate and multivariate analysis, the HACOR scale was the only one that maintained statistical significance. However, when the discrimination capacity of the HACOR and ROX scales was evaluated (Fig. 1), it was found that the AUROC was similar for the two scales without significant differences (ROX AUROC 0.72 vs. HACOR AUROC 0.71). Those were just marginally better than AUROC of RR alone (0.69, 95% CI 0.63–0.76).

Finally, an estimate of the best cut-off point was made using the Liu method to predict the failure of the HNFC, finding that the value for HACOR scale was 5.5 with a sensitivity of 66% and a specificity of 65% (AUROC for the cut-off point of 0.66) while the cut-off point for ROX scale was 5.6 with a sensitivity of 62% and a specificity of 65% (AUROC for the cut-off point of 0.64).

## Discussion

In this study, we investigated the discrimination capacity of the ROX index and the HACOR scale to predict high-flow nasal cannula failure in patients with COVID19 pneumonia. Our data suggest that the discrimination capacity of the 2 scales is similar despite the scales evaluating different variables.

In our study we found that 140 (57%) patients required ETI and 152 (62%) had the final composite outcome of HFNC failure (requirement of ETI or death by COVID19). We believe that the differences in study populations may lead to variation in findings. Compared to the report by Roca et al.^6^, our sample analyzed had a higher median age (64 vs. 53 years). Also, a higher proportion of our patients on HFNC failure had CKD (13.8 vs. 4.5%). Secondly, our study found that patients with atrial fibrillation had a worse outcome. This variable was not evaluated by Roca et al. Furthermore, a later study did find that a heart rate greater than 90 beats per minute was associated with a higher risk of failure of HFNC^19^.

The discriminative capacity of both is similar (ROX AUROC 0.72 vs. HACOR AUROC 0.71). The discriminative capacity of the ROX index is similar to those observed in other studies^12–14^. As for the HACOR scale, so far, we do not know that it has been evaluated in patients with COVID19 infection. In our study, the discriminative capacity of the HACOR scale was similar to that reported by Innocenti et al. (AUROC of 0.68)^20^, but it was lower than that found by Duan et al. (AUROC of 0.89)^10^, and Carrillo et al. (AUROC of 0.88)^12^. We believe that these differences occurred because in the studies by Duan et al., and Carrillo et al. patients with respiratory failure secondary to causes other than COVID19 infection, as well as patients with hypercapnic respiratory failure, were taken. These findings may also be associated because in our study the discriminative capacity of the HACOR scale was taken based on a composite result of the need for ETI and death, similar to the study by Innocenti et al. but different from the Duan study where only the need for ETI was taken as a result. However, more studies are needed to carefully evaluate these hypotheses.

In our study, the best cut-off point for the ROX index to determine the success of HFNC was 5.6 (sensitivity 62% specificity 65%) and not 4.9, as previously established by Roca et al.^11^. On the other hand, the highest cut-off point of the HACOR scale to define the success of the HFNC was 5.5 (sensitivity 66%, specificity 65%), similar to that previously reported in the literature^21,22^.

Interestingly, the HACOR scale was statistically significant in the multivariate analysis when comparing variables that are not included in the calculation of this scale, unlike the ROX index, which did not achieve statistical significance. We believe that this finding is due to the fact that the HACOR scale uses a greater number of variables that reached statistical significance such as the Glasgow scale, pH and PaO2/FiO2 2 h post HFNC, and that are not part of the ROX index. However, the discrimination capacity of the HACOR and ROX scales is similar. Given that the outcome we seek to avoid is mortality, we suggest using both scales to define the failure of the HFNC, which would provide greater safety for the patient, although this would imply a closer monitoring of the person.

Despite the fact that the RR in our study showed a slightly lower performance than the HACOR scale and the ROX index in predicting the failure of the HFNC, we consider that the use of parameters derived from the physical examination of the patient, such as the RR, can be a valuable tool to define the failure to the NIMV and of potential benefit in scenarios with limited resources. In this sense, scales such as WOB scale^23^ have been developed and recommendations such as those of the Chinese Society of Anesthesiology^24^ have been made in using parameters that are derived from simple clinical data such as the RR and the SpO2 to define HFNC failure. However none of them has been tested prospectively to predict HFNC outcome.

Although the predictive capacity of both scales is adequate, we believe that it was not so high that it could be used as the sole criterion to predict failure of the HFNC. Therefore, we consider necessary to add to these tools clinical variables that were also associated with the outcome of interest, such as the presence of viral or bacterial pneumonia concomitant to SARSCoV2 infection. Patients who had coinfection had a higher risk of HFNC failure compared to those who did not (33% vs. 5%). Of the people with the presence of coinfection, the most frequently isolated germs were *Klebsiella pneumoniae* (24%), *S. aureus* (21%), *H. influenzae* (8%) and Influenza B virus (8%). It has been recognised for a considerable time-period, that viral respiratory infections predispose patients to bacterial infections, and that these co-infections have a worse outcome than that of either infection on its own^25^, additionally, previous studies have found that coinfections appear to be associated with the severity of COVID-19 infection and poor outcomes^26,27^.However, The difficulty then is differentiating a patient with a lower respiratory infection, whether the positive respiratory tract test represents carriage or true infection. One large study from the US documented that early empiric antibiotic therapy was used in 57% (965/1705) patients hospitalised with COVID-19, whereas only 4% (59/1705) of patients had a confirmed community-onset bacterial co-infection^27^. These aspects may represent a potential limitation in some of this study.

Additionally, patient's history of HTA and CKD could potentially be useful to predict worse outcomes. These findings are consistent with The OpenSAFELY project^28^. Additionally, the Global Burden of Disease collaboration identified that worldwide, CKD is the most prevalent risk factor for severe COVID-19^29^. Although CKD patients are known to be at increased risk of death due to infectious diseases^30^, the factors contributing to their greater vulnerability for severe COVID-19 should be explored, as these may provide valuable insights into therapeutic approaches to the disease in this patient group. These variables could lead to the development of new models that allow a better prediction of the failure of the HFNC. New studies will be necessary to propose and evaluate these models.

Within the strengths of the study, this is the first study to compare the ability to predict cannula failure of the ROX and HACOR scales. Furthermore, it is one of the individual studies with the largest number of patients evaluated with HFNC and is the first to describe the discriminative capacity of the HACOR scale in patients with COVID19 pneumonia. Finally, we did not have data loss and we were able to count the outcomes of all the patients. Among the limitations of the study is the fact that the criteria to define the ETI requirement depended directly on the treating physician. However, the criteria for therapeutic failure to HFNC are clearly defined in our institution and these are part of a management protocol, which is why we believe that the decision for mechanical ventilation is similar among medical personnel.

## Conclusion

The HACOR scale and the ROX index have a moderate predictive capacity to predict failures to the HFNC strategy. They can be used in conjunction with other clinical variables to define which patients may require invasive mechanical ventilation.
